# A Description of Congenital Anomalies Among Infants in Entebbe, Uganda

**DOI:** 10.1002/bdra.20838

**Published:** 2011-07-18

**Authors:** Juliet Ndibazza, Swaib Lule, Margaret Nampijja, Harriet Mpairwe, Gloria Oduru, Molly Kiggundu, Miriam Akello, Lawrence Muhangi, Alison M Elliott

**Affiliations:** 1 MRC/UVRI Uganda Research Unit on AIDS, Uganda Virus Research InstituteP. O. Box 49, Entebbe, Uganda; 2 London School of Hygiene and Tropical MedicineKeppel Street, London WC1E 7HT, United Kingdom; 3 Entebbe HospitalP.O. Box 29, Entebbe, Uganda

**Keywords:** congenital anomalies, infants, epidemiology, Uganda, Africa

## Abstract

**BACKGROUND:** Data on congenital anomalies from developing countries of the sub-Saharan region are scarce. However, it is important to have comprehensive and reliable data on the description and prevalence of congenital anomalies to allow surveillance and the implementation of appropriate public health strategies for prevention and management. In this study, we describe the profile of congenital anomalies seen in a birth cohort in Entebbe, Uganda. **METHODS:** Congenital anomalies were defined as any structural defect present at birth. Pregnant women were recruited to the cohort between 2003 and 2005. Defects present at birth were recorded by the midwife at delivery and by physicians at the routine six-week postnatal visit and at illness-related visits until 1 year of life. The anomalies were classified by organ system according to the 10th version of the World Health Organization International Classification of Diseases (ICD-10). **RESULTS:** There were 180 infants with a congenital anomaly among 2365 births. The most commonly affected systems were the musculoskeletal (42.7 per 1000 births) and skin (16.1 per 1000 births). The prevalence of major anomalies was 20.3 per 1000 births; 1.7 per 1000 births for cardiac anomalies and 1.3 per 1000 births for neural system anomalies. Forty (22%) of the congenital anomalies were identified at birth, 131 (73%) at the 6-week postnatal visit, and nine (5%) at illness-related visits. **CONCLUSION:** Congenital anomalies are common in developing countries. Establishment of comprehensive databases for surveillance would be helpful for surveillance of effects of new exposures, for prevention, management, and health care planning. Birth Defects Research (Part A) 2011. © 2011 Wiley-Liss, Inc.

## INTRODUCTION

Congenital anomalies are a global problem and every year an estimated 7.9 million children are born with a serious birth defect, 3.3 million children under five years of age die from birth defects, and 3.2 million who survive may be disabled for life (Carmona,^2005^). While more than 90% of all infants with a serious congenital anomaly are born in middle-income and low-income countries, comprehensive data on congenital anomalies in these countries are not available.

Reports have noted the very high prevalence of polydactyly in blacks (Simpkiss and Lowe,^1961^; Kromberg and Jenkins,^1982^) and of the hemoglobin disorders in sub-Saharan Africa (World Health Organization,^1996^) but few reports exist on the prevalence of other congenital anomalies in Africa and it is difficult to compare reports because of differences in study design, methods of ascertainment, and nomenclature. The actual prevalence of congenital anomalies in Africa may be different than in the developed world due to differences in genetics and differences in exposures such as infections, while the recognized prevalence may be different for reasons of underreporting, deficiencies in diagnostic capabilities, and poor follow-up for examination for anomalies in the postnatal period.

In many developing countries, birth defects registries are absent and the health care services, from antenatal through obstetric to postnatal and adolescent health care, are challenged with fundamental gaps in the understanding, prevention, and treatment of congenital anomalies. To date, the only report on congenital anomalies in Uganda is by Simpkiss and Lowe (1961) who described a prospective study done at the national referral hospital between 1956 and 1957. Without comprehensive data on congenital anomalies in this region, it is difficult to evaluate possible teratogens and to institute comprehensive and effective prevention and care services. For example, at this time, with the advent of antiretroviral therapy in pregnant women with HIV/AIDS in Africa, it would be paramount to monitor any effects of antiretroviral therapy used in pregnancy on the incidence of congenital anomalies (Watts,^2007^). In this cohort, data on congenital anomalies were collected carefully, and no association with the trial interventions, albendazole and praziquantel, was found (Ndibazza et al.,^2010^). Therefore, the cohort provides an opportunity to describe the frequency and range of anomalies in a typical African population.

## MATERIALS AND METHODS

### Study Area

This study was part of an ongoing randomized double-blind placebo-controlled trial conducted in Entebbe, Uganda, to determine the effect of helminths and their treatment in pregnancy and in young children on immunologic and disease outcomes in childhood (Elliott et al.,^2007^; Webb et al.,^2011^). The study area comprises Entebbe Municipality and Katabi subcounty, on the Entebbe peninsula in Lake Victoria. The area is occupied by urban, rural, and fishing communities. The population is estimated at 70,200 (Uganda Bureau of Statistics,^2007^), and 93% of deliveries in this community are carried out in health units (Ministry of Health,^2002^). The area is comprised of several tribes; 49% of the pregnant women were from the indigenous Baganda tribe, with less than 10% from any other individual tribe (Woodburn et al.,^2009^).

### Study Population

Consecutive pregnant women were recruited at the Entebbe General Hospital antenatal clinic between April 2003 and November 2005. Women were screened and enrolled in their second or third trimester if they were a resident in the study area, planning to deliver at the hospital, and willing to know their HIV status. Women with severe anemia (hemoglobin <8 g/dL), severe liver disease, history of diarrhea with blood in stool, an abnormal pregnancy, history of adverse reaction to anthelminthics, or who had been enrolled in the study during an earlier pregnancy were excluded. No prenatal screening by ultrasound was carried out.

At enrollment, which was restricted to the second and third trimester of pregnancy, participants were randomized to albendazole or placebo (400 mg; GlaxoSmithKline, Brentford, United Kingdom), and praziquantel (40 mg per kilogram; Medochemie Ltd, Limassol, Cyprus) or placebo, as a single oral dose. All women were offered routine antenatal care, including hematinics (iron and folate), and intermittent presumptive treatment for malaria using sulfadoxine-pyrimethamine. Women were treated for syphilis if a rapid plasma reagin test was positive, and those who tested HIV-positive were provided with nevirapine (200 mg, single oral dose, to be taken at onset of labor) for prevention of mother-to-child HIV transmission.

Written informed consent was obtained from all eligible women. The study received ethical approval from the Science and Ethics Committee, Uganda Virus Research Institute; the Uganda National Council for Science & Technology; and the London School of Hygiene & Tropical Medicine.

Congenital anomalies were defined as any structural defect present at birth. Chromosomal analysis was not done and diagnoses such as Down's syndrome were based on clinical findings. The midwives carried out an external examination for overt anomalies, and no specific examination routine was followed. Anomalies detected by the physicians followed a thorough physical examination, supported when necessary by referral to specialists. Special investigations such as an echocardiogram were only done if there was clinical indication. Infants who had congenital anomalies involving more than one system were recorded once as having a multiple congenital anomaly. When no definite diagnosis was made, the infant was classified and coded as having an unspecified congenital anomaly. The anomalies were classified by organ system according to the 10th version of the World Health Organization International Classification of Diseases (ICD-10; International Classification of Diseases,^2007^).

### Statistical Analysis

Data were analyzed using Stata version 9 (Stata Corp., College Station, TX). The prevalence of congenital anomalies was defined as the number of individual live and stillborn infants with a congenital anomaly per 1000 total births. Associations with maternal age and socioeconomic status, child's sex, multiplicity, and infant mortality were assessed using the chi-square test and logistic regression.

## RESULTS

The flow of the study was previously reported by Ndibazza et al. (2010). There were 2345 live births and 44 stillbirths; 72% were delivered at Entebbe General Hospital, 17% at a private health unit, and 11% at home. Thirty-eight stillbirths (86%) and 2327 live births (99%) were examined for a congenital anomaly from April 2003 to April 2007; 2115 infants were still under follow-up at the age of 1 year. The midwives examined infants delivered at the Entebbe General Hospital; data on findings at delivery were not available for infants delivered elsewhere. The study physicians examined children for congenital anomalies at the six-week postnatal visit or at an illness visit. Forty congenital anomalies (22%) were identified by the midwives at birth, while the physicians identified 131 (73%) at the 6-week postnatal visit and nine (5%) at illness-related visits to the study clinic; an overall prevalence of 76.1 per 1000 births. The prevalence of major anomalies was 20.3 per 1000 births; 1.7 per 1000 births for cardiac anomalies and 1.3 per 1000 births for neural system anomalies. Three of the 38 stillbirths had a congenital anomaly.

Prevalence rates for the different congenital anomalies are shown in Table 1. The most commonly affected systems were the musculoskeletal (42.7 per 1000 births) and skin (16.1 per 1000 births). Among these, defects of the abdominal wall musculature were the most common followed by polydactyly, which often consisted of postaxial polydactyly. One of the infants with defects of the skin had poliosis, while the rest had a birthmark. Only one of the six infants with defects of the genitourinary system was female. Three of the four cases with defects of the cardiovascular system were septal defects, while one had a patent ductus arteriosus. Of the three infants with defects of the neural system, one had anencephaly, another had hydrocephaly, and the third had spina bifida. Of the 10 cases with multiple system defects, five had an umbilical hernia with another system defect, four had a birthmark with another system defect, one had low set ears, a short neck, and a left lower limb deformity, while another had flexion deformity of the fingers on both hands with a macrocephaly and an unspecified anomaly of the gastrointestinal tract. One infant had an unspecified defect of the upper gastrointestinal tract, and another infant had an unspecified anomaly of the respiratory system.

**Table 1:** Classification of Congenital Anomalies According to the ICD-10

System	Number	Percentage	Detail	ICD-10	Anomaly detected at^b^
GIT	1	1	a**Unspecified upper GIT obstruction**	Q 40.9	Illness visit
Genitourinary system	6	3	**Undescended testis & hypospadias**	Q 53.9 & 54.9	Birth
			Phimosis	Q 55.8	Illness visit
			Imperforate hymen	Q 52.3	6 weeks' visit
			Hydrocele (2)	P 83.5	6 weeks' visit
			Small white cyst at base of penis	Q 55.8	Birth
Cardiovascular system	4	2	**Patent ductus ateriosus**	Q 25.0	Illness visit
			**VSD**	Q 21.0	Illness visit
			**Perimembranous VSD**	Q 21.0	Illness visit
			**Atrial-septal defect**	Q.21.1	Illness visit
Dysmorphic	4	2	**Down syndrome**	Q 90.9	Birth
			Facial asymmetry (2)	Q 67.0	Birth/6 weeks'visit
			Short mandible, short maxilla, and high arched palate	Q 38.5	6 weeks' visit
Head & neck	12	7	Small nodular mass, right side of face	Q 82.9	Illness visit
			**Choanal atresia**	Q 30.0	Illness visit
			Ankyloglossia (4)	Q 38.1	6 weeks' visit
			Branchial cleft vestige	Q 18.0	Birth
			Preauricular sinus	Q 18.1	6 weeks' visit
			Preauricular tags (2)	Q 17.0	6 weeks' visit
			Small growth on tongue	Q 38.3	Birth
			Growth right side of neck	Q 18.9	Illness visit
Multiple systems	10	6	Umbilical hernia & ankyloglossia	K 42, Q 38.1	6 weeks' visit
			Inguinal scrotal hernia & birthmark	K 40, Q 82.5	6 weeks' visit
			Preauricular sinus & birthmark	Q 18.1, Q 82.5	6 weeks' visit
			**Umbilical hernia, inguinal hernia & club foot**	K 42, K40, Q 66.0	6 weeks' visit
			**Flexion deformity fingers & microcephaly & unspecified abnormality of the GIT**	Q 68, Q75.3, Q43	Birth
			Umbilical hernia & birthmark (2)	K 42, Q 82.5	6 weeks' visit
			**Low set ears, short neck & left limb deformity**	Q 17.4, Q 18.8,	Birth
			**Umbilical hernia & polydactyly**	Q 74.9	6 weeks' visit
			Umbilical hernia & nodular swelling left frontotemporal	K 42, Q 69.9	6 weeks' visit
				K 42, Q 82.9, Q 55.6, Q 82.5	
Musculoskeletal	101	56	Umbilical hernia (64)	K 42	6 weeks' visit
			Umbilical hernia & inguinal hernia (1)	K 42, K40	6 weeks' visit
			Inguinal hernia (2)	K 40	6 weeks' visit
			**Polydactyly (26)**	Q 69	Birth/6 weeks' visit
			Genu recurvatum (1)	Q 68.2	Birth
			**Congenital club foot (4)**	Q 66.0	Birth/6 weeks' visit
			**Brachymetatarsia (1)**	Q 74.8	6 weeks' visit
			Syndactyly (webbed toes) (1)	Q 70.3	Birth
			**Unspecified lower limb deformity (1)**	Q 74.9	6 weeks' visit
Central nervous system	3	2	**Anencephaly**	Q 00.0	Birth
			**Hydrocephalus**	Q 03.9	Birth
			**Spinal bifida (meningomyelocele & hydrocephalus)**	Q 05.2	Birth
Skin	38	21	Birth mark (37)	Q.82.5	Birth/6 weeks' visit
			Poliosis (1)	Q.84.0	Birth
Unspecified	1	1	**Unspecified anomaly of the cardiorespiratory system**	Q.34.9	Birth
Total	180	100		ICD-10	

a Major anomalies are highlighted in bold; multiple system anomalies that included at least one major anomaly were also classified as major anomalies.

b Anomalies were detected at birth by a midwife, and detected at 6 weeks' or at an illness visit by a study physician.

ICD, International Classification of Diseases Codes; Version 10; GIT, gastrointestinal tract; VSD, ventricular septal defect;

The proportion of total anomalies was higher in boys (8%; 99 of 1224) than in girls (7%; 81 of 1141) but the difference was not statistically significant (*p* = 0.4). Multiple births, maternal age, and socioeconomic status, as well as maternal HIV were not significantly associated with having a congenital anomaly. As previously reported, there was no association between congenital anomalies and the anthelminthic trial interventions (Ndibazza et al.,^2010^).

The congenital anomalies identified among the three stillbirths were anencephaly, polydactyly, and club foot. The infant mortality rate was slightly higher among infants with a congenital anomaly compared to those without a congenital anomaly (odds ratio = 1.81; 95% confidence interval, 0.95–3.46). Hemoglobin disorders are not reported as these were not systematically screened for.

## DISCUSSION

In our study, we recorded an overall prevalence of congenital anomalies, detected between birth and age 1 year, of 76 per 1000 births. The rate of major anomalies was 20.3 per 1000 births. The majority were minor anomalies with minimal effect on clinical function. In an earlier, small study based in the Entebbe Hospital maternity ward, Tann et al. (2005) observed a prevalence of congenital anomalies among neonates of 17 per 1000 births. This much lower figure accords precisely with our observation that only 22% of anomalies were identified by midwives at birth, and could indicate that detection of congenital anomalies may well quadruple from birth to age one year. We observed a slight male preponderance of malformations, similar to findings elsewhere (Lary and Paulozzi,^2001^; Riley and Halliday,^2008^; Bakare et al.,^2009^), and in keeping with the findings of Lary and Paulozzi (2001), we observed that anomalies of the genitourinary system were more prevalent among boys.

We found only one other report on congenital anomalies in Uganda, by Simpkiss and Lowe (1961) who examined 2068 newborn babies for congenital anomalies at the Mulago national referral hospital; this reported an incidence rate of 54 per 1000 births. This rate at birth was higher than ours, which could be a result of more detailed examination at birth or selection bias considering that deliveries at the national referral hospital were more likely high-risk pregnancies. The Simpkiss study, unlike ours, reported a high prevalence of prehelicine fistulas, however, similar to our study, they observed very few anomalies of the neural and cardiac systems, and the prevalence of musculoskeletal system anomalies (excluding umbilical and inguinal hernias) was comparable.

Not everyone would include umbilical and inguinal hernias in congenital anomalies. Both the ICD-9 and ICD-10 classify umbilical and inguinal hernias under ‘Diseases of the Digestive System (K00-K93)’ and not under ‘Congenital malformations, deformations, and chromosomal abnormalities (Q00-Q99)’. However, in clinical practice, umbilical and inguinal hernias are often included among minor congenital anomalies, since they result from the persistence of a weak area in the abdominal muscles. The European Surveillance of Congenital Anomalies, a network of population-based registers for the epidemiologic surveillance of congenital anomalies, also recognizes umbilical hernias as congenital anomalies albeit minor ones. If these anomalies were excluded, the prevalence of musculoskeletal defects in our study population would be 14 per 1000 births. Others have also noted a high prevalence of anomalies of the musculoskeletal system among black populations; 10.07 of 1000 compared to 5.59 of 1000 among the Dutch reference group (Anthony et al.,^2005^), and 27.4 of 1000 compared to 21.2 of 1000 among the white group (Christianson et al.,^1981^). In the same studies, there were relatively lower rates of neural defects; 2.98 of 1000 compared to 3.06 of 1000 among the Dutch reference group (Anthony et al.,^2005^), and 5.4 of 1000 compared to 6.0 of 1000 among the white group (Christianson et al.,^1981^). Hence race and ethnicity should be taken into consideration when the prevalence rates of congenital anomalies are compared in different populations.

In this study, all the cardiac and most of the genitourinary defects were detected in infancy by the research physicians, at the six-week postnatal visit, or at an illness visit. At birth, the midwives detected the more overt anomalies of the neural and musculoskeletal systems. Improvement in the training of midwives in routine examination of the newborn for congenital anomalies, and inclusion of this in their routine duties, is likely to be feasible (Williamson et al.,^2005^) and could particularly improve the recognition and management of important anomalies in settings, such as Uganda, where doctor:patient ratios are very low.

Population-based estimates of the overall prevalence of congenital anomalies and epidemiologic trends are lacking in many African countries. Therefore, birth prevalences of congenital anomalies can only be approximated from hospital-based and community-based data, which may not necessarily be an accurate representation of the general population; although 93% of deliveries in this study area are carried out in health units (Ministry of Health,^2002^), the proportion of births occurring in a health facility in rural Uganda is as low as 36% (Uganda Bureau of Statistics,^2007^). However, in this study, 73% of the congenital anomalies were identified at the six-week postnatal visit, which, according to the national immunization schedule, is the first postnatal visit to a health unit and implies that a considerable number of children born with a birth defect can be identified at this visit, providing more comprehensive data on congenital anomalies in the population. Midwives are usually responsible for care both at delivery and at the 6-week postnatal visit, and would be able to identify overt congenital anomalies but could miss the subtle ones. Sensitization of midwives to the importance of documenting congenital anomalies would improve surveillance for these conditions. Training health professionals to recognize birth defects before hospital discharge would minimize the underestimation of congenital anomalies in this population. Moreover, the introduction of advanced techniques such as fetal visualization using ultrasound screening and chromosome microarray testing at birth, would greatly improve the early detection of anomalies in many developing countries.

Underreporting in our study would have resulted from enrollment of pregnant women in the second and third trimester and exclusion if they had an abnormal pregnancy, a lack of postmortem examination of stillborn infants, and those delivered at home who died in the neonatal period, incomplete follow-up to age one year, and lack of genetic studies for genetic and syndromic defects, or of the hemoglobinopathies; all of these may have resulted in an underestimation of the overall prevalence of congenital anomalies. However, our study provides a fair representation of data on congenital anomalies in a typical African population.

## CONCLUSION

Even as extensive literature is available on congenital anomalies in the developing world, there is a dismal scarcity of comparable data from Sub-Saharan Africa. As developing countries continue to see a decline in childhood infectious diseases, congenital anomalies will take on a greater significance as a cause of morbidity and mortality. Training of midwives in surveillance for congenital anomalies and their management should be encouraged for appropriate prevention, management, and health care planning.
